#  Exon Deletion Pattern in Duchene Muscular Dystrophy in North West of Iran 

**Published:** 2015

**Authors:** Mohammad BARZEGAR, Parinaz HABIBI, Mortaza BONYADY, Vahideh TOPCHIZADEH, Shadi SHIVA

**Affiliations:** 1 Pediatric Health Research Center, Tabriz University of Medical Sciences, Tabriz, Iran.; 2 Division of Pediatric Neurology, Tabriz Children’s Hospital, Tabriz University of Medical Sciences, Tabriz, Iran; 3 Center of excellence for biodiversity, Department of Molecular Medical Genetics, Faculty of Natural Sciences University of Tabriz, Tabriz, Iran; 4 Physical Medicine & Rehabilitation Research Center, Tabriz University of Medical Sciences, Tabriz, Iran

**Keywords:** Dystrophin, Multiplex PCR, Duchenne Muscular Dystrophy, Becker Muscular Dystrophy

## Abstract

**Objective:**

Duchene and Becker Muscular Dystrophy (DMD/ BMD) are x-linked disorders that both are the result of heterogeneous mutations in the dystrophin gene. The frequency and distribution of dystrophin gene deletions in DMD/ BMD patients show different patterns among different populations. This study investigates the deletion rate, type, and distribution of this gene in the Azeri Turk population of North West Iran.

**Materials &Methods:**

In this study, 110 patients with DMD/ BMD were studied for intragenic deletions in 24 exons and promoter regions of dystrophin genes by using multiplex PCR.

**Results:**

Deletions were detected in 63 (57.3%) patients, and around 83% localized in the mid-distal hotspot of the gene (on exons 44–52), 21 cases (33.3 %) with single-exon deletions, and 42 cases (66.6%) with multi-exonic deletions. The most frequent deleted exons were exon 50 (15 %) and exon 49 (14%). No deletion was detected in exon 3.

**Conclusion:**

This study suggests that the frequency and pattern of dystrophin gene deletions in DMD/ BMD in the Azeri Turk population of North West Iran occur in the same pattern when compared with other ethnic groups.

## Introduction

 Duchenne (OMIM 310200) and its less severe allelic form Becker (OMIM 300376) muscular dystrophy (DMD/ BMD) are common x-linked recessive hereditary neuromuscular diseases. These disorders result from heterogeneous mutations in the dystrophin gene (1-2). The incidence of DMD is 1 in 3500 and BMD is 1 in 18000 male births (3-4). The dystrophin gene that spans a distance of more than 2.5 million base pairs is located at the Xp21 locus and is the largest human gene, which consists of 79 exons. Sequencing this huge gene is time-consuming and, fortunately, the deletions in this gene are non-randomly distributed with many of the large gene deletions that occur in the dystrophin gene can be detected in specific hotspot areas of the gene. These hotspots are clustered in two main regions: 1. about 20% at the 5’ proximal portion of the gene (exons 1,3,4,5,8,13,19); and 2. 80% at the mid-distal region i.e. 42–45, 47, 48, 50–53 (5-6). 

The reading frame rule explains the clinical differences between DMD/ BMD at the molecular level with the deletions that cause shifts in the reading frame of the dystrophin mRNA (out-of-frame) that lead to no or very low production of functional dystrophin resulting in more severe DMD phenotype whereas the milder BMD phenotype occurs if the reading frame is preserved (in-frame) and semi functional proteins are produced (7). In about 65% of DMD cases, the mutation of the dystrophin gene are large-scale gene deletions, in approximately 5% are duplications and in 30% are point mutations (8- 10). Due to no effective treatment available for DMD/ BMD at present, an accurate genetic diagnosis may offer prenatal diagnosis for familial DMD/ BMD although about 30% of cases are the result of new mutations (11). 

The frequency and distribution of dystrophin gene deletions in DMD and BMD patients show that there are different patterns among different populations. In a previous study of authors in East Azerbaijan Province, in 46 male patients with clinical suspicion of DMD/ BMD, 21 (46%) had deletions. 

This study investigates the rate, types, and distributions of deletions in DMD/ BDM patients (with a relatively large sample) in the Azeri Turk population of North West Iran by using multiplex polymerase chain reaction (MPCR) assays. 

## Materials & Methods

All patients were evaluated by an expert pediatric neurologist during 2004–2013. Clinical diagnosis in 104 patients was made by using standard clinical diagnosis criteria for DMD/ BMD in conjunction with high serum levels of Creatine Phosphokinase (CPK) at least 10 times the upper limit of normal and compatible electromyographic results (12). In 6 patients, the diagnosis was confirmed by a muscle biopsy. 

All patients were of Azeri Turk origin (or from North West Iran). Patients (or their parents) were informed about the study and written consent was signed by either the patient or the parents for blood sampling. This study was approved by Tabriz University of Medical Sciences Ethical Committee. Genomic DNA was extracted from peripheral blood leukocytes using standard protocols. 

For molecular genetic studies, MPCR assays were done using three complementary MPCR assays that detected 26 exons of the dystrophin gene (6). Three separate PCR assays were performed on each patient’s DNA sample to amplify 26 dystrophin gene exons (13). The following exons were studied during this protocol: exon number: 3, 4, 6 , 8, 12, 13, 16, 17, 19, 32, 34, 41, 42 , 43, 44, 45, 46, 47, 48, 49, 50, 51, 52, and 60; and the promoter zone. 

## Results

**Fig 1:**
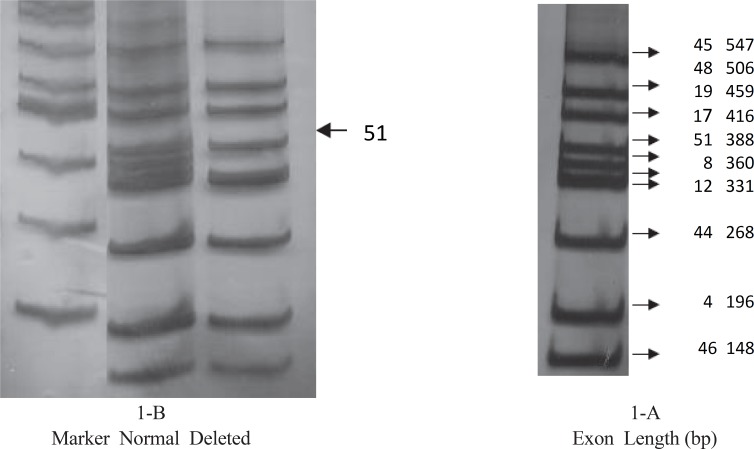
Each sample was studied for 24 exons to detect possible exon deletion. Line 1: Marker- Line 2: Normal sample- Line 3: Sample from DMD patient showing deletion in exon 51

Each patient’s DNA sample was checked alongside a normal sample for 24 exon-deletion (Fig. 1) 

**Fig. 2:**
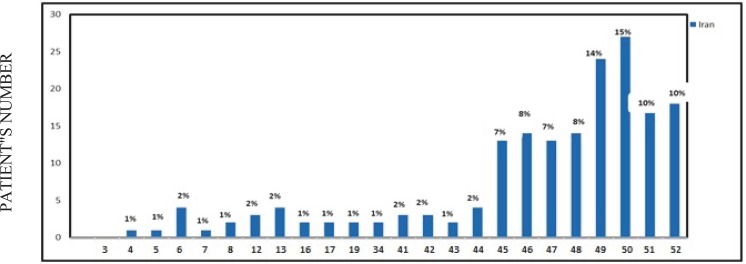
Distribution of gene deletion in 24 exons in patients with DMD/ BMD in North West Iran

In this study, deletions were detected in 63 (57.3%) patients, among these 21 patients (33.3%) had single exon deletions and 42 patients (66.7%) had more than one deletion (range 2–8): 20 patients (31.7%) had two deletions, 4 patients (6.3%) had three deletions, 6 patients (9.5%) had four deletions, 4 patients (6.3%) had five deletions, 1 patient (1.6%) six deletions, 3 patients (4.8%) had seven deletions, and the remaining 4 patients (6.3%) had 8 exon deletions. The most frequently deleted exons were exon 50 (15%) and exon 49 (14%). No deletion was detected on exon 3. Approximately 83% of all deletions were located on exons 42–52 mid-distal hot spot (Fig. 2). 

In this study, patients with Becker totaled 6, and considering the diversity of distribution for gene deletions in these patients. Because of the small sample size, statistical analysis to determine genotype and phenotype correlations was impossible. 

## Discussion

Duchenne and Becker muscular dystrophy are common inherited recessive x-linked motor diseases. Mapping and molecular genetic studies have indicated that both are the result of mutations in the huge gene that encodes dystrophin. There are various laboratory techniques to assess deletion of the dystrophin gene. Some studies have used the Southern Blot technique for this purpose, which is difficult and time consuming. Nevertheless, it has applications in identifying deletion and duplication of the whole gene, locating the origin of deletions and duplications to determine the effect of mutation on the reading frame, and, ultimately, to find the exon junctions. A Southern blot permits prognostication of severity by distinguishing in-frame versus frameshift mutations in over 90% of cases. Some studies have used MPCR, which are cheaper, faster, and easier than Southern Blot; and because of its high speed, it is ideal for prenatal diagnosis. However, it can only allow for the identification of deletions. Therefore, it is advised to concurrently run both tests. Another method is the use of quantitative PCR (QPCR) to determine gene dosage. This method has an important application in determining carriers, but deleted or duplicated exons should first be identified (14-16). 

**Tabel 1:** Deletion Pattern in DMD/ BMD in 19 Different Countries

**Country**	**Total** **number of Candidates** **examined**	**Total** **Number and percentage** **of deletions**	**Deletions on the proximal region of gene** **(Number and percentage** **of deletion)**	**Deletions on the mid-distal region** **(Number and percentage** **of deletion)**
Bulgaria	183	122(66.67)	18(14.75)	97(79.51)
Denmark	196	101(51.53)	25(24.75)	77(76.24)
France	103	45(43.69)	13(28.89)	32(71.11)
Hungary	159	116(72.96)	20(17.24)	91(78.45)
Italia	294	211(71.77)	40(18.96)	157(74.41)
The Netherlands	361	327(90.58)	63(19.27)	230(70.34)
UK	552	273(60.53)	46(16.85)	217(79.49)
Canada	346	219(63.29)	22(10.04)	159(45.95)
USA	550	392(71.27)	98(25.0)	271(69.13)
China	205	109(53.17)	33(30.28)	74(67.89)
Philippines	35	11(31.43)	7(63.64)	4(36.36)
India	332	223(67.17)	33(14.80)	187(83.86)
Japan	217	113(52.07)	23(20.35)	55(48.67)
Turkey	242	146(60.33)	23(15.75)	120(82.19)
Argentina	174	86(49.43)	30(34.88)	52(60.47)
Brazil	251	235(93.63)	56(23.83)	159(67.66)
Chile	51	24(47.06)	2(8.33)	21(87.5)
Egypt	152	78(51.32)	15(19.23)	59(75.64)
Australia	481	279(58.00)	66(23.66)	192(68.82)
Present Study (Iran, Tabriz)	110	63(57.3)	30(17)	145(83)

The Multiplex Ligation-dependent Probe Amplification (MLPA) technique allows for the possibility to assess all deletions or duplications of the dystrophin gene. Lai et al. compared MPCR and MLPA and concluded that MLPA detected all known deletions and duplications as well as the extent and distribution of the deletions and duplications, which in turn facilitated a genotype-phenotype correlation (17). 

According to this study, using MPCR in Azeri Turk patients in North West Iran, showed that intragenic deletions were detected in 63 (57.3%) DMD/ BMD patients. 

There are various reports on the frequency and pattern of intragenic deletions in different populations. According to these reports, in addition to deletion frequency differences in different populations, the distribution of deletions in the dystrophin gene is different. Table 1 presents a summary of rate and distribution patterns of deletion for different countries available in the Leiden Muscular Dystrophy database (http://www.dmd.nl/ DMD_deldup.html). 

Table 1 shows that in North America and Europe, the deletion rates vary from 42% to 90%, deletion rates in Asian countries vary from 31% to 86%, and the deletion in Latin American countries varied from 47% to 93%. The deletion rate in Africa was reported at 51%. 

In this study, 83% of deletions were in the mid-distal hotspot of the gene (exons 44–52) and 17% in the 5’ proximal portion of the gene (exons 4–22). The highest frequency of deletion was observed in exon 50 (15%), then followed by exon 49 (14%). 

Table1 shows that European countries varied between 70–80% of deletions and are located in central areas of dystrophin gene (exons 44–52). In North America, 45– 70% of deletions are in the central area. In most Asian countries, deletions are in the center area of gene as well.

However, the population of the Philippines shows that most deletions are at the beginning of the gene. The same results are reported from other countries as well (18-20). In this respect, a few studies have been conducted in Iran, including a study by Akbari et al. on 100 patients with Duchenne and Becker dystrophy with 52 patients (52%) showed deletion, 81% of deletions were at the end (exons 44–55), and 19% at the first hotspot of gene (exons 2–19). The majority of deletions were identified in exons 47, 48, and 46 (18-21). 

In Galehdari et al. reported that in Ahwaz 17 patients with suspected Duchenne gene deletion was present in 53% of patients, all of which were in exons 44–51 (19-22). In a previous study, 46 boy patients in East Azerbaijan Province showed that 21 patients (46%) had deletions. The majority of deletions were observed in exons 45, 49, and 50 at a frequency of 14.3%. These exons were present as the most mutationable exons in patients in this province (20-23). 

In our study, the rate and distribution of deletions in patients was similar to the deletion pattern in previous studies in Iran and in terms of pattern of deletion and distribution Iran and Turkey are similar (21-24). 

The difference in distribution of deletions can be explained by inclusion criteria for diagnosis of cases, for example: biopsy confirmed or clinical suspicion, different methods used for genetic examination i.e. MLPA technique, or cDNA, Southern Blot or multiple PCR or combination of them; and number of exons studied in MPCR. Finally, the variations strongly suggest that sequence differences exist in the introns and that the differences are in agreement with genetic distances among populations. The similarities further suggest that some intronic sequences have been conserved and that those will trigger recurrent deletions (22-25). 

In this study, there were 6 patients with Becker, and considering the diversity of the distribution of gene deletion in these patients, and because of the small sample size, statistical analysis was impossible. However, the results of various studies have shown that based on gene deletion patterns, the disease phenotype cannot be determined (23-27). 

Among the applied objectives of this study was to enable research groups and patients to participate in new clinical trials through the identification of gene deletion patterns and based on these indicators and genetic mutations. Researchers are working on exon skipping as a method to introduce novel dystrophin production and turn severe DMD into BMD type disease (i.e. a milder form). As mentioned earlier, DMD is caused by mutations in the DMD gene, mostly the deletion of one or more of the 79 exons in the dystrophin gene. These mutations disturb the synthesis of the dystrophin protein. The specific skipping of one or more exons flanking the specific deletion in DMD patients allows the introduction of BMD-like dystrophin and as a result, converts severe DMD into a typically milder BMD. The exon-skipping approach is mutation specific. Therefore, each mutation needs a separate exon to be skipped and compound to induce it. Currently, new therapeutic strategies, such as antisense-mediated exon skipping, are in early phase of clinical trials and have the potential to change the course of the DMD disease dramatically. In one study, intramuscular injection of an antisense oligonucleotide (AON) induced skipping of exon 51 and restored the disrupted open reading frame and, therefore, the production of dystrophin in 4 DMD patients with deletion of exons 48–50, 49–50, 50, and 52, respectively. Clinical trials with systemic administration of AON are taking place, and, if successful, therapeutic skipping using an AON that targets exon 51 can stop further muscle wasting to result in a clinical phenotype like BMD. This AON can be applied to about 13% on the DMD patients. (28-30). 

**In conclusion, **the present study suggests that the frequency and pattern of dystrophin gene deletions in DMD/ BMD in the Azeri Turk population in North West Iran occur with the same pattern compared with other ethnic groups. 

## Notes

**Fund: **This study was supported by the Tabriz University of Medical Sciences
